# Editorial: Surviving and thriving: how crops perceive and respond to temperature stress, volume II

**DOI:** 10.3389/fpls.2025.1757791

**Published:** 2026-01-08

**Authors:** Zemin Wang, Yi Wang, Darren Chern Jan Wong

**Affiliations:** 1 State Key Laboratory of Aridland Crop Science, Gansu Agricultural University, Lanzhou, China; 2 College of Life Science and Technology, Gansu Agricultural University, Lanzhou, China; 3 State Key Laboratory of Efficient Production of Forest Resources, Yinchuan, China; 4 Beijing Key Laboratory of Grape Science and Enology, Chinese Academy of Sciences (CAS) Key Laboratory of Plant Resources, Institute of Botany, Chinese Academy of Sciences, Beijing, China; 5 School of Agriculture, Food, and Wine, Waite Research Institute, Adelaide University, Urrbrae, SA, Australia

**Keywords:** agroecosystem stability, climate-smart agriculture, crop resilience, flowering time, heat and cold stresses, molecular adaptation, phenology shifts, temperature stress

Climate change is reshaping global agroecosystems, with extreme heat and cold events emerging as major constraints on crop productivity and food security (Zhu, 2016; Jin et al., 2024; Mittler et al., 2025). Understanding how plants perceive and adapt to temperature anomalies has never been more urgent. This second volume of *Surviving and Thriving: How Crops Perceive and Respond to Temperature Stress* advances that mission, exploring molecular and physiological mechanisms underpinning resilience. Building on the success of the first volume (Wang et al., 2025), which featured 12 original studies across cereals, legumes, vegetables, and fiber crops, this edition adds eight new contributions. These studies broaden the phylogenetic scope to include an alternative oilseed, a cereal, a tropical perennial, and an emerging fiber nettle crop, deepening our understanding of temperature stress adaptation across diverse plant lineages (**Figure 1**).

**Figure 1:**
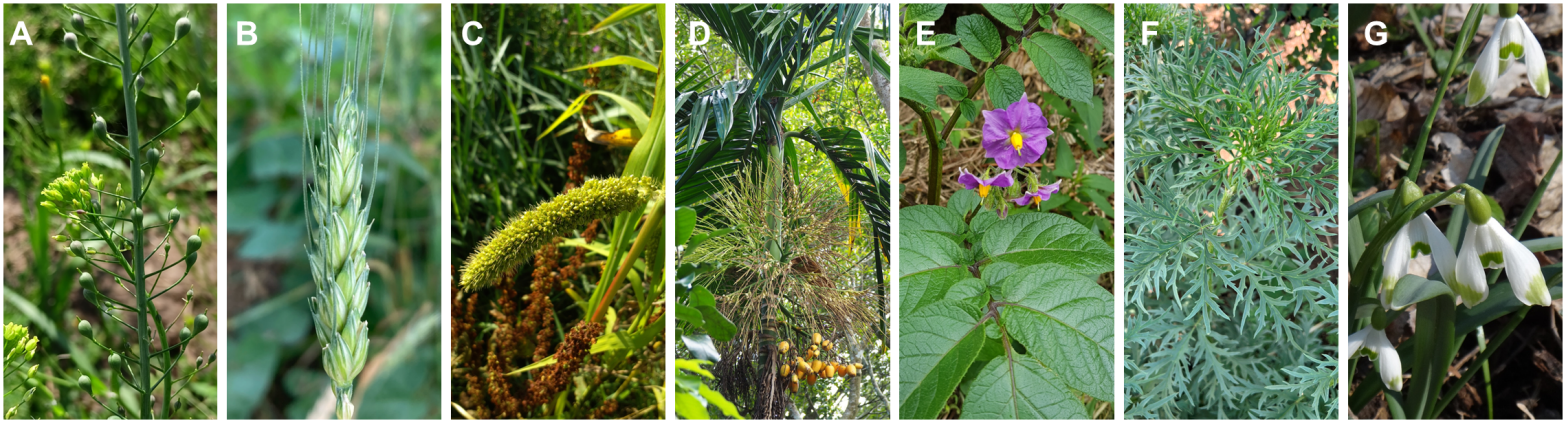
An Illustration of phylogenetic breadth, highlighting major, emerging and underutilized species across global agroecosystems featured in this topic. **(A)** Gold-of-pleasure (*Camelina sativa*, Brassicaceae), **(B)** Wheat (*Triticum aestivum*, Poaceae) **(C)** Foxtail millet (*Setaria italica*, Poaceae) **(D)** Areca palm (*Areca catechu*, Arecaceae) **(E)** Potato (*Solanum tuberosum*, Solanaceae) **(F)** Siberian hemp nettle (*Urtica cannabina*, Urticaceae) **(G)** Snowdrop (*Galanthus nivalis*, Amaryllidaceae). All images were sourced and adapted from iNaturalist under the Creative Commons CCO 1.0 Universal Public Domain Dedication. No additional permissions were required.

Beginning in the Mediterranean basin, where prolonged summer heat increasingly coincides with reproduction, *Camelina sativa* has become a model for studying thermal stress at critical reproductive stages (**Figure 1A**). Alberghini et al. investigated two cultivars by imposing a five-day heat treatment at the end-of-flowering (*Exp1*) and end-of-silique formation (*Exp2*). *Exp1* affected growth and yield, while *Exp2* influenced oil quality and tocopherol accumulation. Both stresses reduced oil content, but late stress likely triggered an antioxidant surge implicated in membrane and photosystem stabilization. This response involved trade-offs, as oil content fell by 10–12% and fatty acid profiles shifted, suggesting diversion from storage lipids to protective mechanisms. Future breeding efforts should prioritize genotypes that withstand stage-specific heat stress common in Mediterranean climates, while maintaining high yield and robust nutritional quality.

Moving northeast into the temperate wheat belt, genomic dissection of CONSTANS-like transcription factors (COL) has become central to understanding resilience in wheat (**Figure 1B**). Gao et al. catalogued 51 *COL* genes in wheat, clustered them into three subfamilies with conserved domains, and assessed their evolutionary conservation across grasses (Poaceae). Integrated analyses spanning promoter motif landscapes, expression profiles, co-expression networks, and functional assays converge on Ta-5D-COL16, a nuclear-localized activator that ties flowering progression to environmental adaptation. As photoperiod and temperature patterns shift, *COL* genes offer precise targets to fine-tune flowering and stress resilience without sacrificing yield.

South and inland into semi-arid Asian drylands, foxtail millet (*Setaria italica*) faces episodic cold that undercuts productivity (**Figure 1C**). Yang et al. prioritized a cold-responsive SiCST1, a nuclear protein with a ribonuclease H-like domain, by comparative transcriptomics. Cross-species validation encompassing CRISPR/Cas9 knockout of the rice homolog severely impaired cold tolerance, an effect reversed by complementation, underscoring functional conservation. Protein interaction assays place SiCST1 alongside SiOFP1 (an OVATE family protein) within a brassinolide (BR) signaling. A mechanistic model where cold-induced SiCST1 modulates BR pathway components to rebalance growth-defense trade-offs, stabilizing cellular function when temperatures dip was put forth. For highland and steppe margins where planting windows are narrow, BR-focused modulation offers a route to cold-hardy millet.

Crossing the South China Sea to Hainan Island, the tropical palm *Areca catechu* reveals how a warmth-adapted species copes with rare chills (**Figure 1D**). Thriving at 20-25°C, occasional drops to 6.8 °C exposes *A. catechu* to cold damage in the region. Li et al. combined physiological and transcriptomic analyses to reveal a biphasic cold acclimation: an early ROS surge activates peroxidases, while chlorophyll levels stabilize, indicating photosystem protection rather than collapse. Thousands of cold-responsive genes were identified. Network analysis identified 25 modules and six hub genes linked to chromatin remodeling, lipid signaling, and cell-wall restructuring, offering transcriptomic insights that provide subtropical plant breeders a roadmap for marker-assisted selection and microclimate management.

Across temperate zones, the world’s staple potato (*Solanum tuberosum*) is increasingly exposed to rising thermal loads, threatening yield stability (**Figure 1E**). Two consecutive studies by Zhu et al. identified two Group II WRKY factors, StWRKY65 and StWRKY75, as central integrators of thermotolerance. Overexpression of either *StWRKY65* and *StWRKY75* improve vegetative growth and tuber yield, elevates chlorophyll, or enhances photosynthetic metrics (photosynthesis, transpiration, stomatal conductance), while reducing H_2_O_2_ and malondialdehyde (two key molecular signatures of oxidative damage). StWRKY75 further upregulates heat shock proteins, reinforcing proteostasis. Knockdown lines of *StWRKY65* and *StWRKY75* also exhibited opposite effects confirming their crucial roles. For northern latitudes facing episodic heat stress, breeding strategies targeting *WRKYs* offer a route to sustain canopy photosynthesis and tuber development.

East across continental interiors, winter-prone plains test the limits of *Urtica cannabina*. In these areas, nutrient management emerges as a critical layer of defense (**Figure 1F**). Liu et al. showed that nitrogen and phosphorus fertilization not only mitigates freezing injury but also enhances antioxidant enzyme activity, elevates soluble sugars and proline to maintain osmotic balance, and limits oxidative damage during prolonged low-temperature exposure. Multi-omics integration points to modulation of flavonoid and phenylpropanoid biosynthetic pathways that provide ROS-scavenging metabolites while network analysis highlights hub gene modules that can be co-targeted by breeding and nutrient regimes. In regions with otherwise occasional cold spells during otherwise mild winters, nutrient-mediated priming may help strengthen plants beyond genetic improvements.

Ultimately, validation of resilience requires testing under field conditions where crops encounter natural environmental variability. Hauser et al. presented an innovative low-cost, semi-controlled field warming platform that elevates temperatures while preserving natural variability. Validated on Arabidopsis and tomato, and notably on snowdrop (*Galanthus nivalis*), a threatened ornamental species on the global International Union for Conservation of Nature Red List (**Figure 1G**), this platform enables phenotypic assessment (e.g. flowering time) under realistic diurnal and seasonal dynamics. This innovative platform, with detailed construction plans and software code, supports climate adaptation studies in major crops like wheat, while enabling research on underutilized species in remote and resource-limited environments.

## Summary

These contributions deepen our understanding of how plants respond to temperature stress across diverse agroecosystems. As climate change intensifies, failure to adapt or migrate will drive local plant extinctions, with altered flowering times disrupting pollination services and triggering cascading effects that undermine ecosystem stability and resilience (Christmas et al., 2016; Wong et al., 2023). This underscores the urgency of integrating molecular insights with field-ready innovations to build climate-resilient agroecosystems, secure food supplies, and protect biodiversity.
